# Muscle oxygen consumption by NIRS and mobility in multiple sclerosis patients

**DOI:** 10.1186/1471-2377-13-52

**Published:** 2013-05-29

**Authors:** Anna Maria Malagoni, Michele Felisatti, Nicola Lamberti, Nino Basaglia, Roberto Manfredini, Fabrizio Salvi, Paolo Zamboni, Fabio Manfredini

**Affiliations:** 1 Program Pathophysiology of Vascular Peripheral System, S. Anna Hospital University of Ferrara, Cona, Via A. Moro, 8, Ferrara, 44124, Italy; 2 Vascular Diseases Center, University of Ferrara, Ferrara, Italy; 3 Department of Rehabilitation Medicine, S. Anna Hospital, Ferrara, Italy; 4 Clinica Medica, Department of Medical Sciences, University of Ferrara, Ferrara, Italy; 5 Center for Rare and Neuroimmunological Diseases, Department of Immunological Science, Bellaria Hospital, Bologna, Italy

**Keywords:** Multiple sclerosis, Muscle metabolism, Non-invasive, Near-infrared spectroscopy, Oxygen consumption

## Abstract

**Background:**

The study of muscle metabolism by near-infrared spectroscopy (NIRS) has been poorly implemented in multiple sclerosis (MS). Aims of the study were to compare resting muscle oxygen consumption (rmVO_2_) at gastrocnemius in MS patients and in age-matched healthy controls (HC) measured using NIRS, and to evaluate its possible relationship with patients’ mobility.

**Methods:**

Twenty-eight consecutively enrolled MS patients (male, n = 16; age = 42.7 ± 14.0 y, Relapsing-Remitting, n = 19; Primary-Progressive, n = 9) and 22 HC (male, n = 13; age = 36.0 ± 8.2 y) were studied during rest applying the NIRS probes at gastrocnemius, producing a venous occlusion at the thigh using a cuff, and analyzing the slope of the total hemoglobin to calculate rmVO_2._ Mobility was assessed by a 6-Minute Walking Test and 6-Minute Walking Distance (6MWD) was recorded.

**Results:**

rmVO_2_ was higher in MS compared to HC (0.059 ± 0.038 vs 0.039 ± 0.016 mlO_2_/min/100 g, P < 0.003), not different in clinical subtypes, not correlated to patients’ characteristics (age, disease duration, Expanded Disability Status Scale, resting heart rate, skinfold thickness), and significantly higher in patients with lower walking ability (6MWD < 450 m, n = 12) compared to those at better performance (respectively, 0.072 ± 0.043 vs 0.049 ± 0.032 mlO_2_/min/100 g, P = 0.03).

**Conclusion:**

rmVO_2_ values, significantly higher in MS patients compared to HC, and in low versus high performing patients, might represent a marker of peripheral adaptations occurred to sustain mobility, as observed in other chronic diseases.

## Background

Multiple Sclerosis (MS) is a neurological disorder characterized by inflammatory demyelination and neurodegeneration within the central nervous system. This condition leads to a variety of symptoms, among which reduced mobility, weakness and fatigue are common and key problems [1,2], apparently due to alterations in both central motor drive and intramuscular function [2]. The study of muscle metabolism in MS by Near-Infrared Spectroscopy (NIRS) has been poorly implemented. NIRS is a technique that allows the non-invasive study of muscle metabolism in static and dynamic conditions in health and disease [3-9]. A parameter that can be easily measured by NIRS is the local resting muscle oxygen consumption (rmVO_2_) [10], which allows a quantification of the muscle’s capacity to extract oxygen from blood. This parameter was found to be impaired in legs of patients with chronic diseases [5,7,8,11,12], and modified following exercise training in peripheral arterial disease (PAD) [13]. Therefore, rmVO_2_ might be potentially useful in a clinical setting for assessing the level of skeletal muscle metabolic impairment, and for detecting muscle modifications following the progression of the disease, therapeutic treatments or rehabilitative programs. We hypothesized that muscle adaptations might also occur and be detectable in MS.

This cross-sectional study aims: i) to compare the rmVO_2_ values at gastrocnemius collected on a sample of MS patients and healthy controls (HC), and evaluate possible differences in MS clinical subtypes; and ii) to evaluate possible relationships between rmVO_2_ values and MS patients’ mobility.

## Methods

### Subjects

Twenty-eight MS patients referred from a regional MS were consecutively enrolled (male, n = 16; age = 42.7 ± 14.0 y). Inclusion criteria: adult patients with MS clinically defined according to the McDonald criteria [14]. Exclusion criteria: Expanded Disability Status Scale score (EDSS) > 6 [15], comorbidities affecting oxygen transport, delivery and extraction (e.g. severe anemia, PAD, etc.), acute relapse within the previous 30 days.

Twenty-two healthy age-matched adults were randomly recruited among rehabilitation laboratory staff (male, n = 13; age = 36.0 ± 8.2 y). All participants gave written informed consent. The study was approved by the ethics committee of Ferrara, Italy.

### Resting muscle oxygen consumption by Near-Infrared Spectroscopy

NIRS measurements were obtained with a continuous wave system (Oxymon-MK III, Artinis Medical Systems, Netherlands) providing measures of changes in oxy- and deoxyhemoglobin concentrations. This system, which consists of 2 channels (2 equivalent pulsed light sources, 2 avalanche photodiode detectors, shielding from ambient light), uses intensity-modulated light at a frequency of 1 MHz and laser diodes at 3 wavelengths (905, 850, and 770 nm) corresponding to the absorption wavelengths of oxy- and deoxyhemoglobin, with an autosensing power supply (approximately 40 W at 110–240 V). The light from the laser diodes is conducted from the instrument to the tissue and back along 3m-long optical glass fibers.

After measuring adipose tissue thickness (ATT) according to international standards [16] along the medial aspect of gastrocnemius muscle, NIRS sensors were placed at the same level. The interoptode distance was maintained at 4 cm, allowing a maximum penetration depth of light around 20 mm. As previously reported [12], rmVO_2_ was measured with the subject resting in a supine position by rapidly inflating a cuff placed around the thigh to a pressure of 60 mmHg to obtain venous occlusion. The pneumatic cuff was quickly released after 30 seconds. The absolute rmVO_2_ value was calculated by the rate of increase in concentrations once the venous outflow had been blocked [17]. Data collection and calculation were performed using the software Oxysoft 47 (Artinis Medical Systems, Netherlands).

During NIRS evaluation, resting heart rate (rHR) was recorded by a heart rate monitor (Sport Tester-RS400, Polar Electro-Oy, Kempele, Finland) and the mean value was considered for the analysis. Measurements were obtained by the same operators.

### Mobility assessment

MS patients were asked to perform a 6-min walking test (6MWT), considered as a feasible, reproducible, and reliable measure providing sensitive information about the walking performance of persons with MS [18,19]. Patients were instructed to walk up and down a 22 m corridor at their own pace for 6 minutes, aiming to cover as much distance as possible. The distance completed after 6 minutes (6MWD) was recorded.

### Statistical analysis

Data are expressed as means ± standard deviations. The normal distribution of the data was verified by the Kolmogorov-Smirnov test. Comparison of demographics was performed using unpaired Student T-tests and Fisher’s exact test, as appropriate. Differences between subgroups were measured by an unpaired Student *T*-test or One-way ANOVA test, as appropriate. A Pearson correlation was performed to evaluate the relationship between rmVO_2_ values in MS patients and possible influencing variables (age, disease duration, rHR, ATT). A Pearson correlation was also performed to evaluate a possible relationship between rmVO_2_, and EDSS, Pyramidal Functions (PF) subscale score of Kurtzke Functional Systems Scores [15], 6MWD, and rHR.

Analysis was performed considering rmVO_2_ values of both legs, and the mean value of the two legs of each subject, when appropriate.

Significance was set at a P-value ≤ 0.05. Statistics were performed using MedCalc 12.4.0.0 (MedCalc Software, Mariakerke, Belgium).

## Results

### Study population

Among the MS population, 19 patients were a Relapsing Remitting (RR) clinical type, and 9 were Primary Progressive (PP). PP patients were older than HC and RR (P < 0.001). Characteristics of study participants are presented in Table 1.

**Table 1:** Demographics of study participants

	**All MS (n = 28)**	**RR (n = 19)**	**PP (n = 9)**	**HC (n = 22)**
**Sex (n)**	M = 16; F = 12	M = 10; F = 9	M = 6; F = 3	M = 13; F = 9
**Age (years)**	42.7 ± 14.0	34.2 ± 7.6	60.3 ± 4.3^†^	36.0 ± 8.2
**Disease duration (years)**	9.9 ± 6.3	7.4 ± 3.3	14.4 ± 8.5	–
**EDSS**	2.7 ± 1.6	1.9 ± 1.0	4.3 ± 1.3	–

MS, Multiple Sclerosis; RR, Relapsing Remitting; PP, Primary Progressive; HC, healthy controls; EDSS, Expanded Disability Status Scale.

### Resting muscle oxygen consumption values in MS patients and healthy controls

ATT and rmVO_2_ were safely measured in both legs for all MS (n = 56) and HC (n = 44). ATT was less than 20 mm for all participants.

**Figure 1:**
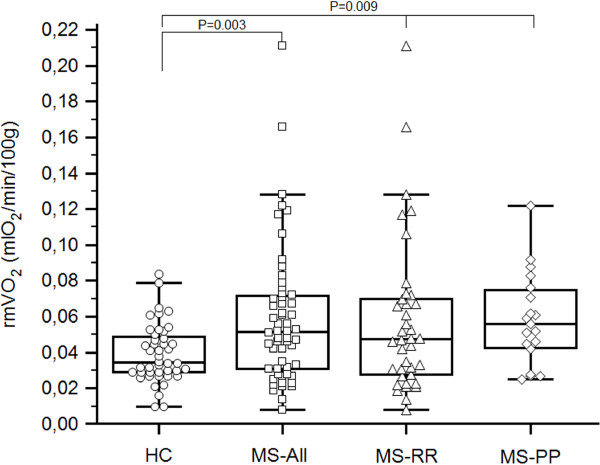
**Comparison between rmVO**_**2 **_**values of legs of healthy and MS subjects.** Legend to figure: rmVO_2_, resting muscle oxygen consumption; HC, healthy controls, MS, Multiple Sclerosis; RR, Relapsing Remitting; PP, Primary Progressive. Statistical analysis: Unpaired Student *T*-test between HC and all MS; One-way ANOVA among HC, RR, and PP.

rmVO_2_ values of both legs resulted in being significantly higher in all MS compared to healthy (P = 0.003), even separately RR and PP (P = 0.009) (Table 2, Figure 1). PP showed the highest rmVO_2_ values, but no significant difference with respect to RR and HC.

**Table 2:** Values of main parameters recorded in study participants

	**All MS (n = 28)**	**RR (n = 19)**	**PP (n = 9)**	**HC (n = 22)**
**Legs (n)**	56	38	18	44
**rmVO**_**2 **_**(mlO**_**2**_**/min/100g)**	0.059 ± 0.038*	0.058 ± 0.043^†^	0.060 ± 0.025^†^	0.039 ± 0.016
**rHR (beats/min)**	85 ± 10*	83 ± 11	89 ± 8	79 ± 6

MS, Multiple Sclerosis; RR, Relapsing Remitting; PP, Primary Progressive; HC, healthy controls; rmVO_2_, resting muscle oxygen consumption; rHR, resting heart rate.

*P < 0.05 by Unpaired Student *T*-test between all MS and HC;

^†^ P < 0.05 by One way-ANOVA among RR, PP, and HC.

Not significantly higher rmVO_2_ values (mean of the two legs for each subject) were observed in patients with EDSS ≤ 2 (n = 14) compared to patients with EDSS >2 (n = 14) (respectively 0.051 ± 0.029 vs 0.067 ± 0.040 mlO_2_/min/100 g, P = n.s.). A similar pattern was depicted comparing rmVO_2_ values of patients categorized in two groups according to the PF subscale score, more strictly related to muscular functions (PF ≤ 1, n = 16 vs PF > 1, n = 12, respectively 0.051 ± 0.027 vs 0.069 ± 0.043 mlO_2_/min/100 g, P = n.s.).

rHR was significantly higher in all MS patients compared to HC (P = 0.02) (Table 2).

No significant relationship was found in all MS patients, and in clinical types separately, between EDSS and rmVO_2_ values (mean of the two legs for each subject), as well as considering the PF subscale score (mean value = 1.8 ± 1.1).

No significant relationships were found between the rmVO_2_ values of all MS patients, considering possible influencing factors (age, disease duration, rHR, ATT), even evaluating clinical types separately. In all MS a trend towards a direct relationship between rHR and rmVO_2_ (mean of the two legs for each subject) (r = 0.37, P = 0.065) was observed.

### Resting muscle oxygen consumption and mobility

All MS patients performed the 6MWT. The mean value of 6MWD was 489.5 ± 194.0 m.

On the basis of the mean walking speed of healthy individuals of the same age (1.26 m/sec) [20], patients were categorized in two groups, a group of lower walking ability (n = 12, PP, n = 9, RR, n = 3) and a group of better performance (n = 16, RR, n = 16, PP, n = 0), considering a cut-off in 6MWD equal to 450 m.

**Figure 2:**
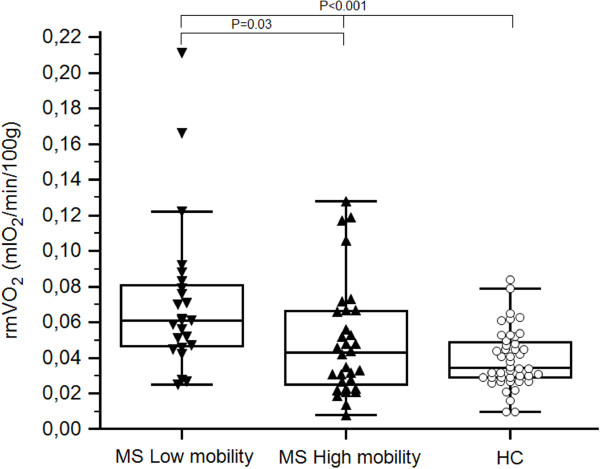
**Comparison between rmVO**_**2 **_**values of legs of MS population ranked according to mobility. Values of healthy controls were also included.** Legend to figure: rmVO_2_, resting muscle oxygen consumption; MS, Multiple Sclerosis; HC, healthy controls. Statistical analysis: Unpaired Student *T*-test between MS low and high mobility; One-way ANOVA among MS low and high mobility, and HC.

The group of lower walking ability showed significantly higher rmVO_2_ values of both legs compared to the group of better performance (P = 0.03) (Table 3), and healthy controls (P < 0.001) (Figure 2). As additional observation, the same significant difference was highlighted considering the rHR (P < 0.004) (Table 3). Furthermore, in the entire MS population, 6MWD was also found inversely correlated to rHR (r = −0.44, P = 0.02), whilst no correlation was found with rmVO_2_ values (mean of the two legs for each subject).

**Table 3:** Comparison between the two subgroups of MS patients according to walking performance

	**MS Low performance (6MWD < 450 m) (n = 12)**	**MS High performance (6MWD > 450 m) (n = 16)**	**P value**
**Legs (n)**	24	32	
**6MWD (m)**	294.3 ± 106.8	636.6 ± 70.5	<0.0001
**rmVO**_**2 **_**(mlO**_**2**_**/min/100g)**	0.072 ± 0.043	0.049 ± 0.032	=0.03
**rHR (beats/min)**	91 ± 9	81 ± 9	<0.004

MS, Multiple Sclerosis; 6MWD, 6 Minute Walking Distance; rmVO_2_, resting muscle oxygen consumption; rHR, resting heart rate.

## Discussion

The present study, to the best of our knowledge, for the first time deals with the measurement of rmVO_2_ by NIRS in MS patients. This parameter determined by venous occlusion was found to be significantly higher in MS patients than in healthy subjects, in absence of differences between clinical subtypes, EDSS and PF subscale scores, and with no correlation to patients’ age, disease duration, rHR, ATT, EDSS, and PF subscale score. Interestingly, low performing patients showed higher rmVO_2_ values compared to better performing subjects, as well as a higher resting heart rate.

The rmVO_2_ parameter was also studied in some other patients with chronic diseases, such as chronic heart failure (CHF) [11] and PAD [12]. Lower rmVO_2_ values at brachioradialis muscle have been observed in patients with CHF compared to healthy subjects [11]. Just in another population with walking impairment as PAD patients, compared to healthy subjects, did we observe rmVO_2_ values at gastrocnemius by venous occlusion, which were significantly higher in more severely diseased patients [12], similar to those observed in the present MS population. In that case a possible compensatory mechanism to the lack of oxygen has been hypothesized. To support this hypothesis of plastic peripheral adaptations in the O_2_ system, different rmVO_2_ values were observed in the legs of PAD patients after a 6-month exercise program, with increased values in limbs without hemodynamic improvements and lower changes (decrease) in the limbs less ischemic following rehabilitation [13].

In MS patients where blood flow restrictions are not present, an abnormal pattern of muscle fiber composition and size with respect to healthy people has been observed, with fewer type I fibers, a shift towards white fibers, and smaller fibers of all types [2]. This picture seems to be consistent with the pattern described in subjects exposed to deconditioning [2]. In a group of chronic stroke survivors, values of rmVO_2_ superimposable to those observed in MS patients were observed in the hemiplegic leg, with a decrease to reach the lower values of the unaffected limb after 10 weeks of over-ground gait training (unpublished data).

Therefore, it seems that a pathological (neurologic or vascular) condition plus a deconditioning phase is responsible for an adaptive increase of this parameter.

However, skeletal muscle energetic status during rest does not seem significantly impaired in MS compared to healthy subjects, with no indication of metabolic inflexibility or mitochondrial dysfunction [21], thus with a likely normal adaptive attitude. Our results in MS patients might be explained assuming that the residual type I fibers develop a greater metabolic activity and enhanced capillarization as a compensatory mechanism to sustain walking endurance capacity and fatigue. Otherwise, variation in artero-venous difference is a factor influencing rmVO_2_ together with the blood flow, per se, potentially supported by the higher rHR observed in MS patients, and more in lower performing ones, compared to HC.

This preliminary study, with its limitations due to the small sample size and NIRS technique (limited region of muscle evaluated, variability of probe position, ATT, etc.), sheds some light on non-invasive muscle evaluation by NIRS in MS. rmVO_2_ at gastrocnemius, a quick and painless measurement suitable for a clinical setting [12], might represent a biomarker of peripheral adaptations related to patients’ mobility. It was found to be stable in repeated measurements in normally active, healthy subjects, and modified following 6-month training in PAD patients with a different adaptive response according to the hemodynamic changes observed in each leg [13]. Along these lines, investigations into rmVO_2_ might be an interesting issue in MS, for the purpose of monitoring rehabilitative programs. In the present study, we focused on gastrocnemius, based on previous experience [12,13], and its relation to mobility. However, other muscle groups can also be measured to observe whether changes in rmVO_2_ occur in MS during the progression of the disease or in response to treatments.

## Conclusions

rmVO_2_ values measured by NIRS were found to be significantly higher in MS patients compared to HC, and in low versus better performing patients. Such parameter might represent a marker of peripheral adaptations occurred to sustain mobility. It might be potentially useful in a clinical setting for assessing the level of skeletal muscle metabolic impairment, and for monitoring the progression of the disease, therapeutic treatments or rehabilitative programs.

## Abbreviations

ATT: Adipose tissue thickness; EDSS: Expanded disability status scale score; HC: Healthy controls; MS: Multiple sclerosis; NIRS: Near-infrared spectroscopy; PAD: Peripheral arterial disease; PF: Pyramidal functions subscale score; PP: Primary progressive; rHR: Resting heart rate; rmVO2: Resting muscle oxygen consumption; RR: Relapsing remitting.

## Competing interests

The authors declare that they have no competing interests.

## Authors’ contributions

AMM, conceived and designed the study, collected the data, analyzed and interpreted the data, drafted the manuscript; MF, collected the data, analyzed and interpreted the data; NL, collected the data, analyzed and interpreted the data; NB, participated in the design of the study and revised the manuscript; RM, participated in the design of the study and revised the manuscript; FS, participated in the design of the study and revised the manuscript; PZ designed the study, analyzed and interpreted the data, and critically revised the manuscript; FM, conceived and design the study, analyzed and interpreted the data, drafted and critically revised the manuscript. All authors read and approved the final manuscript.

## Pre-publication history

The pre-publication history for this paper can be accessed here:

http://www.biomedcentral.com/1471-2377/13/52/prepub
